# Macroscopic Dynamic Modeling of Sequential Batch Cultures of Hybridoma Cells: An Experimental Validation

**DOI:** 10.3390/bioengineering4010017

**Published:** 2017-02-23

**Authors:** Laurent Dewasme, François Côte, Patrice Filee, Anne-Lise Hantson, Alain Vande Wouwer

**Affiliations:** 1 Automatic Control Department, University of Mons, 7000 Mons, Belgium; alain.vandewouwer@umons.ac.be; 2 Biotechnology Department, CER Groupe, 6900 Aye, Belgium; f.cote@cergroupe.be (F.C.); p.filee@cergroupe.be (P.F.); 3 Department of Chemical and Biochemical Engineering, University of Mons, 7000 Mons, Belgium; anne-lise.hantson@umons.ac.be

**Keywords:** Mathematical modelling, monoclonal antibody, bioprocess optimization, maximum likelihood principal component analysis

## Abstract

Hybridoma cells are commonly grown for the production of monoclonal antibodies (MAb). For monitoring and control purposes of the bioreactors, dynamic models of the cultures are required. However these models are difficult to infer from the usually limited amount of available experimental data and do not focus on target protein production optimization. This paper explores an experimental case study where hybridoma cells are grown in a sequential batch reactor. The simplest macroscopic reaction scheme translating the data is first derived using a maximum likelihood principal component analysis. Subsequently, nonlinear least-squares estimation is used to determine the kinetic laws. The resulting dynamic model reproduces quite satisfactorily the experimental data, as evidenced in direct and cross-validation tests. Furthermore, model predictions can also be used to predict optimal medium renewal time and composition.

## 1. Introduction

Therapeutic products (vaccines, antibodies, etc.) are subject to exponential demands and cost-lowering process improvements, leading to the intensification of growth conditions in the bio-pharmaceutical industry and the sharp increase of the related market economy. For instance, monoclonal antibody (MAb) market amounts to several billion dollars and is still increasing.

To improve bioprocess yield and repeatability, monitoring and control tools are required. The latter implies the availability of dynamic models, which can predict the process trajectory and support the design of software sensors or control strategies. Previous optimization studies of hybridoma cell cultures for MAb production were usually conducted using simple mathematical models based on macroscopic reaction schemes such as in [1,2].

More recently, a macroscopic model with kinetics accounting for overflow metabolism, where glucose and glutamine are the main substrates, was proposed in [3]. Indeed, cell respiratory capacity is limited [4]. Therefore, depending on the substrate concentrations, cell metabolism follows two possible pathways: the respirative regime if the respiratory capacity is sufficient to oxidize the whole substrate amount or the respiro-fermentative regime if this substrate amount is in excess with respect to the available oxidative capacity, thus leading to the formation of growth-inhibiting by-products (respectively lactate from glucose and ammonia from glutamine). The main drawback of such complex kinetic models is however the relatively large number of parameters and the necessity to achieve experiments in specific conditions so as to trigger the phenomena (activation, saturation or inhibition) reflected in the model. 

A more practical alternative is to directly deduce the model structure from the available data, assuming that the data sufficiently cover the process operating range. In [5], Principal Component Analysis (PCA) is used to determine the minimum number of reactions required to interpret the data. This methodology was further extended in [6], where an insightful geometric interpretation is provided, and maximum likelihood principal component analysis (MLPCA) is used to estimate the reaction number and stoichiometric matrix. In this study, the latter approach is applied to the culture of hybridoma cells in sequential batch reactors (SBR). This mode of operation is common in industrial practice, and poses the question of the information content of data sets collected during the several batches. Before embarking in the analysis of actual experimental data, synthetic data are first generated with a process simulator, with the possibility of varying the initial conditions of the batches and the data sampling rate within each single batch. The methodology is subsequently applied to real-life experimental data, with a better grasp of the data information content.

The main contribution of this study is threefold:

- A simple dynamic model of cultures of hybridoma cells in SBR is developed and validated with experimental data. Confidence intervals for the parameters and the estimated trajectories are provided.
- A systematic model identification procedure, based on rigorous yet simple to use tools—MLPCA to determine the stoichiometry, nonlinear least squares to identify the parameters of the kinetic laws, sensitivity analysis and Monte Carlo analysis to infer the confidence intervals—is assessed in a real case study, showing good performance and promise for future applications.
- The simple dynamic model is further exploited to optimize the medium renewal strategy in the sequential batches.

This paper is organized as follows. Section 2 reviews the basic concepts of verflow metabolism and mathematical modeling using principal component analysis. Section 3 presents the experimental case study and process operating conditions. The dynamic model of hybridoma sequential batch cultures is derived in Section 4 and parameters are identified from experimental data in Section 5. Subsequently, Section 6 develops a parametric sensitivity analysis and proposes further model simplifications. The simplified model is identified and cross-validated with two different data sets in Section 7. Finally, the dynamic model is used to optimize the culture medium renewal time and composition in Section 8 while conclusions are drawn in Section 9.

## 2. Dynamic Modeling of Hybridoma Cultures 

This section first reviews the basic concept of overflow metabolism and then presents a systematic procedure to infer candidate macroscopic models from principal component analysis of the data at hand.

### 2.1. Overflow Metabolism

The main physiological feature of hybridoma resides in their primary metabolism or, more precisely, in their catabolism, presenting the following main pathways:

- The glycolysis which is a series of degradation reactions of glucose (the main substrate) taking place in the cytoplasm and leading to a final product, i.e., pyruvate.
- The Krebs cycle, also called the tricarboxylic acids (*TCA*) cycle or citric acids cycle, which takes place inside the mitochondrions and uses pyruvate to product the cells energy units (Adenosine triphosphate or *ATP*) and reduced cofactors (typically *NADH* and *FADH*).
- The electron transport, still taking place in mitochondrions and producing *ATP* from the reduced cofactors.
- The fermentative pathway which, in oxygen limitation, produces typical products like lactate from pyruvate in the cytoplasm.

Cell catabolism is characterized by a limited energy production (i.e., the Krebs cycle has a limited capacity) principally used for cell growth and division. This limitation comes from the capacity to oxidize the main nutrients: glucose (main carbon source) and glutamine (main nitrogen source). The excess amounts of these nutrients are assumed to follow other metabolic pathways more commonly known as “fermentation”, producing a side byproduct.

This “Overflow Metabolism” or “short-term Crabtree effect” [4,7,8,9,10,11], is typically observed with yeast, bacteria and animal cell cultures. Depending on the case, it leads to the production of ethanol, acetate and lactate/ammonium as side byproducts. Several descriptions of this switching mechanism have been proposed in the literature (for instance in [10]) but this phenomenon remains not well understood.

The byproduct formation usually inhibits the oxidative capacity of the cells, slowing down growth for increasing concentrations. In turn, it depends on the oxidative capacity of the cells and on the medium composition. 

A generic mechanistic model that would, in principle, allow the representation of the culture of different strains presenting overflow metabolism, can be described through the following main reactions:

*j*th substrate consumption:

(1a) $$k_{Si}S_{j}\overset{\phi_{S}}{\to }k_{Xi}X+k_{Pi}P_{j}$$

*j*th substrate overflow:

(1b) $$k_{Si+1}S_{j}\overset{\phi_{Over-S}}{\to }k_{Xi+1}X+k_{Pi+1}P_{j}$$

*j*th byproduct consumption:

(1c) $$k_{Pi+2}P_{j}\overset{\phi_{P}}{\to }k_{Xi+2}X$$

where *X*, *S_j_* and *P_j_* are the concentrations of cell biomass, *j*th substrate and *j*th side byproduct, respectively. The *k_ni_* coefficients represent the yield (or pseudo-stoichiometric) coefficients of component *n* in reaction *i*. Overflow metabolism assumption involves that, for each concerned substrate, these reactions take place in pairs (1a and 1b) or triplets (1a–1c) if the considered byproduct can be reconsumed by the biomass as a substitute substrate source when the oxidative capacity is not fully exploited.

Indeed, Sonnleitner and Käppeli [12] assume that the cell oxidative capacity rules the general metabolism, following a bottleneck effect. During a culture, the cells are likely to change their metabolism depending on the exploitation of the respiratory capacity. At low substrate uptake rate (substrate concentrations are below critical levels *S* < *S_crit_* and *φ_S_* < *φ_Smax_*), substrate is consumed with biomass growth and a relatively low metabolite byproduct production (1a) without overflow, which is defined as respiratory metabolism and the consequent remaining respiratory capacity can be used to oxidize byproduct as substitute carbon source as in (1c). 

**Figure 1:**
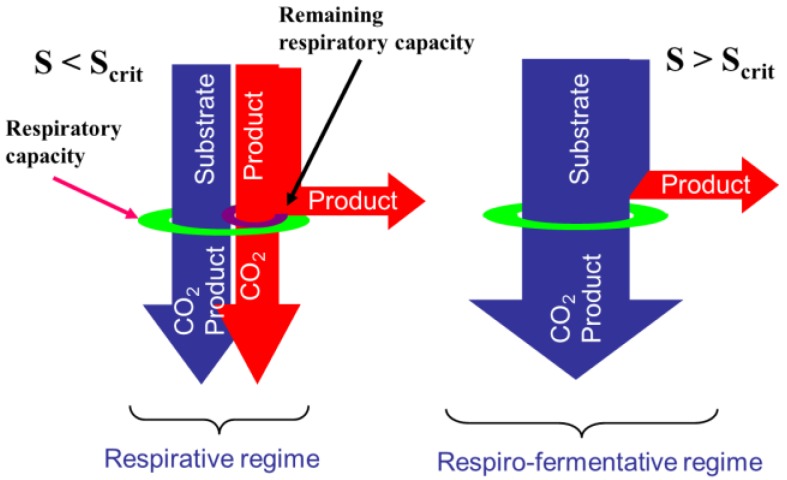
Schematic representation of the bottleneck assumption of Sonnleitner and Käppeli [12].

At high substrate uptake rate (substrate concentration is above critical level *S* > *S_crit_* and *φ_S_* > *φ_Smax_*), the respiratory capacity is saturated, resulting in overflow metabolism towards excess metabolite production (reactions (1a) and (1b)). The state at which overflow metabolism is initiated (*S* = *S_crit_* and *φ_S_* = *φ_Smax_*) is referred to as critical metabolism. For instance, yeast metabolism is described by the bottleneck assumption of Sonnleitner and Käppeli [12], as illustrated in Figure 1.

This model was exploited in [13,14,15] for robust control purposes. Based on a similar model, references [16,17] suggested practical ways to estimate state variables such as biomass, glucose or acetate in bacteria cultures using software sensors. Recently, reference [3] proposed a dynamic model of hybridoma fed-batch cultures based on a double overflow mechanism, one linked to glucose, and the other to glutamine, with good prediction capabilities. 

### 2.2. Systematic Modeling Procedure

In contrast with the previous modeling approach which is based on past experience and a priori knowledge of the metabolic network, it is now suggested to derive a model based mostly on the information content of available data sets. This can be particularly relevant when the model structure is uncertain and experimental data sets are available that can be analyzed to extract information on the reaction stoichiometry and kinetics.

First, we recall that bioprocesses can be represented by macroscopic reaction schemes involving *M* reactions between *N* components under the following generic form [18]:

(2) $$\sum_{i\in {ℜ}_{j}}(-k_{ij})\xi_{i}\overset{\phi_{j}(\xi ,\vartheta_{j})}{\to }\sum_{i\in {℘}_{j}}(k_{ij})\xi_{j}$$

where ${ℜ}_{j}$ (respectively, ${℘}_{j}$) denotes the set of reactants (or products) in the “*j*th” reaction. The parameters *k_ij_* are pseudo-stoichiometric coefficients while $\phi_{j}$ is the corresponding reaction rate.

Applying mass balances to (2), the following ordinary differential equation system is obtained:

(3) $$\frac{d\xi (t)}{dt}=K\phi (\xi ,\vartheta )+\upsilon (\xi ,t)$$

where *K* is the pseudo-stoichiometric matrix and υ represents the transport term taking dilutions, input feeds and gaseous outflows into account. ϑ is the vector containing all the kinetic parameters. 

The number of components *N* is generally larger than the number of reactions *M* so that the rank of the stoichiometric matrix *K* is assumed to be *M*. For instance, in [3], *M = 5* and *N = 6*. 

Defining the transport-free state evolution $\xi_{f}$ and integrating (3) between two consecutive measurement times lead to the following expression:

(4) $$\xi_{f_{i}}^{\Delta }=K\int_{t_{i}}^{t_{i+1}}\phi (\tau )d\tau$$

where $\xi_{f_{i}}^{\Delta }$ is the differential transport-free state vector. As discussed in [6], Equation (4) expresses that $\xi_{f_{i}}^{\Delta }$ is contained in a *M*-dimensional linear subspace, and MLPCA allows to determine subspaces of increasing dimensions *p* explaining a noisy data set (and therefore reaction schemes of increasing detail explaining the experimental data). A systematic procedure can therefore be developed, which selects the smallest value of *p* that allows a thorough interpretation of the data up to a given confidence level, minimizing a log-likelihood cost:

(5) $$J_{p}=\sum_{i=1}^{n_{S}}(\xi_{f,m_{i}}^{\Delta }-{\hat{\xi }}_{f}^{\Delta ,p}{)}^{T}{\left(Q_{i}^{\Delta }\right)}^{-1}(\xi_{f,m_{i}}^{\Delta }-{\hat{\xi }}_{f}^{\Delta ,p})$$

where *n_s_* is the number of measured vector samples and $\xi_{f,m_{i}}^{\Delta }$ is the noisy measurement of $\xi_{f}^{\Delta }$, with an error covariance matrix $Q_{i}^{\Delta }$ and ${\hat{\xi }}_{f}^{\Delta ,p}$ is its maximum-likelihood (ML) estimate by the reduced p-dimensional linear model [6]. *Jp* is a decreasing function of *p* which is always smaller or equal to the log-likelihood cost J* of the true nonlinear model. Since J* is known to have a chi-square distribution with $n_{S}xN$ degrees of freedom [19]. The number of reaction is just chosen as the smallest *p* such that the log-likelihood cost *Jp* is smaller or equal to the range of a χ${}_{n_{S}xN}^{2}$-distributed random variable.

Once the number of reactions is determined, the resulting *N* by *p* affine subspace basis $\hat{\rho }$ can be used to estimate a stoichiometric matrix $\hat{K}$, which is a linear combination of the basis vectors, i.e.,

(6) $$\hat{K}=\hat{\rho }G$$

with *G* a *p* by *p* regular matrix. 

For a complete estimation of the stoichiometry, *p* biological constraints have to be imposed in each column of $\hat{K}$ (for instance the fact that a specific reactant or product is involved in only one reaction).

## 3. Experimental Case Study—Materials and Methods

### 3.1. Operating Conditions

In the framework of this study, six sequential suspended hybridoma batch cultures of 2 hybridoma strains (called, for the sake of confidentiality, HB1 and HB2) were performed in two series of three 200 mL T-flasks. In this protocol, at the initial time of each batch, biomass is kept in the reactor, while the metabolites (lactate, ammonia and monoclonal antibodies) are withdrawn and the substrate concentrations (glucose and glutamine) are set to prescribed values (respectively ranging between 6 and 7 g/L, and 0.3 and 0.4 g/L). The end-of-batch viable and dead biomass concentrations are considered as the initial conditions of the next batch (the initial biomass concentration of the first batch is 0.1 × 10^6^ cells/mL). The culture time is approximately 15 days and one medium renewal is performed approximately after one week. Measurements are taken once every day.

The culture medium is based on 10% FBS (ThermoFischer, Waltham, MA, USA) added to DMEM (Lonza, Belgium) with 6 g/L of glucose and 4 mM of l-glutamine, and is replaced at a specific time (approximately after one week) when one of the substrates (glucose and glutamine) is exhausted, in order to avoid starving. Most of the times, due to the selected medium composition, glutamine is the limiting substrate. As glucose measurement can be performed relatively quickly with respect to the other analytical methods, medium refreshments are achieved based on glucose concentration evolution. Indeed, when glutamine vanishes, glycolysis stops and glucose is not oxidized anymore. Once this phenomenon is observed, the medium is replaced within the day. 

Concerning the culture basic parameters, pH medium is set between 7.2–7.6 at the beginning of the batch and decreases to a minimum of pH between 6.7 and 7.0. The temperature is regulated at 37 °C in a 5% CO_2_ incubator.

### 3.2. Measurements and Data Sets

Measurements are collected off-line following different methods with respect to the component/analyte:

- Biomass: Living and dead biomasses are measured by cell-counting using Trypan blue and a Neubauer chamber.
- Glucose concentration is measured by using a Roche glycemic analytical device called Accu-Chek allowing a fast calculus of the glucose concentration within a few seconds.
- Lactate concentration is also measured using a Roche device called Accutrend delivering fast concentration measurements using dipsticks.
- A “Mega-Calc” enzymatic kit from Megazyme is used to obtain the glutamine and ammonium concentrations. This method is based on absorbance measurements.
- Antibody concentration is obtained using an ELISA dosage of murine IgG designed by the CER group from Aye (Belgium) based on reactants from Bethyl Laboratories (ref A90-131A for coatage antibodies and A90-131P for revelation).

**Figure 2:**
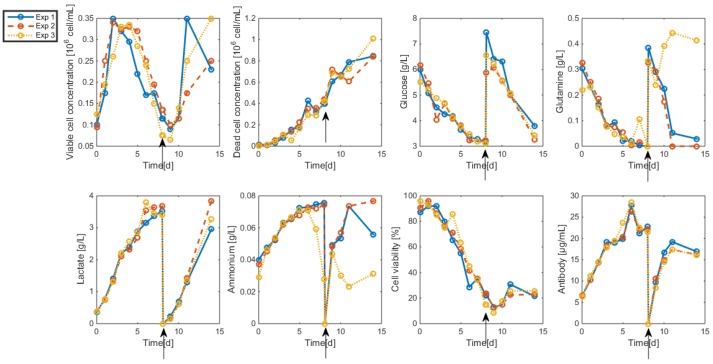
HB1 sequential batch culture data sets. Medium refreshment times are indicated by the arrows.

**Figure 3:**
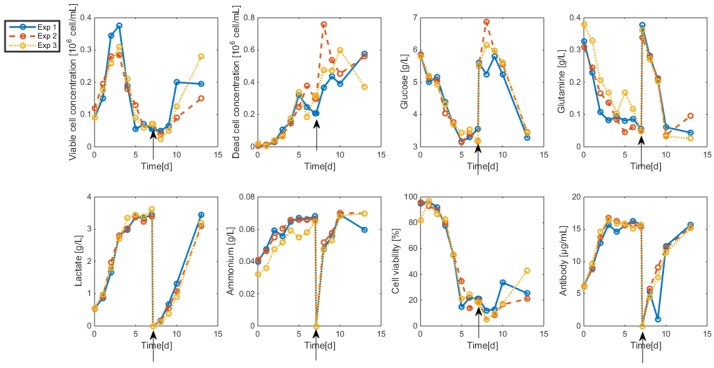
HB2 sequential batch culture data sets. Medium refreshment times are indicated by the arrows.

The resulting data are shown in Figure 2 and Figure 3. As apparent, cell viability decreases significantly after four days but is maintained around 30% thanks to the medium replacement. The level of ammonium concentration is very low and ranges below the sensitivity level of the measurement method. Therefore, ammonium is not considered in the modeling study since concentrations are far below the growth-inhibiting level. Only the glucose overflow, producing lactate, will be taken into account.

## 4. Data-Driven Model Derivation

### 4.1. Data Processing

Before applying MLPCA to the data sets, elimination of data outliers should be achieved in order to reject measurement inconsistencies. For instance, the last increasing glutamine concentration measurements of HB1 third experiment should not be used in identification (and direct validation) since glutamine production is not possible. 

Even if part of the data is discarded for identification, all the measurements can be considered in cross-validation. In the next sections, the first two sets of HB1 data are selected for identification, and the rest of data for cross-validation.

### 4.2. MLPCA-Based Systematic Procedure

The methodology presented in Section 2.2 is now applied to the first two data sets of HB1.

**Figure 4:**
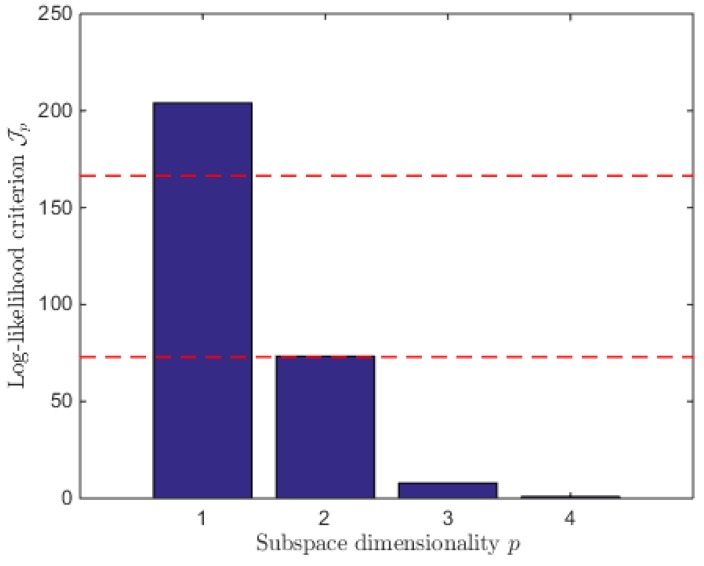
Log-likelihood costs of the p-dimensional subspaces of the first two HB1 data sets. The upper dashed line represents the chi-square quantile χ${}_{28x6}^{2}$ at 99.9% and the lower one the chi-square quantile χ${}_{28x6}^{2}$ at 0.1%.

As shown in Figure 4, a 3-dimensional subspace (i.e., *p* = 3 reactions) is sufficient to interpret the data.

The following matrix ρ represents the maximum likelihood principal components defining the subspace basis related to Figure 4:

(7) $$\rho =\left(\begin{array}{ccc} -0.0074 & 0.0317 & 0.4314\\ -0.0173 & -0.0045 & -0.3108\\ 0.1366 & -0.5955 & -0.6581\\ 0.0169 & -0.0404 & -0.0561\\ -0.1389 & 0.7778 & -0.5300\\ -0.9805 & -0.1940 & -0.0153 \end{array}\right)$$

To obtain a biologically-consistent stoichiometric matrix, reaction constraints have to be expressed so as define a matrix G as introduced in Equation (6):

(a) The existence of a glycolysis pathway where biomass grows on substrates, producing no lactate and without mortality (${\hat{k}}_{11}=1$, ${\hat{k}}_{21}=0$, ${\hat{k}}_{51}=0$);
(b) A sole glucose overflow pathway, according to the absence of ammonium (i.e., of glutamine overflow), where no dead biomass nor antibody is produced (${\hat{k}}_{12}=1$, ${\hat{k}}_{22}=0$, ${\hat{k}}_{62}=0$);
(c) A biomass death pathway (${\hat{k}}_{13}=-1$, ${\hat{k}}_{23}=1$) theoretically with no substrate or metabolite concentration variations. The latter would represent too many constraints with respect to the available degree of freedom and arbitrarily, only the lactate coefficient is set to zero (${\hat{k}}_{53}=0$). Indeed, due to the size of G, which is a 3 by 3 matrix, only 3 constraints can be expressed per reaction.

The general constrained problem can be summarized as:

Find $g_{ij}\in ℜ$ solving (6),

(8a) i,j∈{1,…,M}

*s.t.*

(8b) $$k_{lj}\in \left\{-1,0,1\right\},\;l\in \left\{1,\ldots ,N\right\}$$

In contrast with [5,6], this case study offers the possibility to explore the scenario where biomass is produced out of several macro-reactions.

A specific $\hat{K}$ matrix related to the constrained problem (8) is provided by (9):

(9) $$\hat{K}=\left(\begin{array}{ccc} 1 & 1 & -1\\ 0 & 0 & 1\\ -3.2892 & -19.5802 & 1.9307\\ -0.5219 & -1.3757 & 0.0349\\ 0 & 25.4828 & 0\\ 36.1432 & 0 & 16.4965 \end{array}\right)$$

Apparently, the glucose and glutamine stoichiometric coefficients in the third reaction (i.e., ${\hat{k}}_{33}$ and ${\hat{k}}_{43}$) are small compared to the sum of their respective values in reactions 1 and 2. A possible scenario is therefore to consider that ${\hat{k}}_{33}$ and ${\hat{k}}_{43}$ could be set to zero (the coefficient deviations is partly explained by the lack of information in the data and the measurement noise).

The corresponding reaction scheme becomes:

Substrate oxidation:

(10a) $$\left|{\hat{k}}_{31}\right|G+\left|{\hat{k}}_{41}\right|Gn\overset{\phi_{1}}{\to }X+{\hat{k}}_{61}MAb$$

Substrate overflow:

(10b) $$\left|{\hat{k}}_{32}\right|G+\left|{\hat{k}}_{42}\right|Gn\overset{\phi_{2}}{\to }X+{\hat{k}}_{52}L$$

Biomass death:

(10c) $$X\overset{\phi_{3}}{\to }X_{d}+{\hat{k}}_{63}MAb$$

where $\phi_{1}$, $\phi_{2}$ and $\phi_{3}$ are the reaction rates introduced in Section 5.1.

The corresponding mass balance equations are:

(11a) $$\frac{dX}{dt}=(\phi_{1}+\phi_{2}-\phi_{3})X$$

(11b) $$\frac{dX_{d}}{dt}=\phi_{3}X$$

(11c) $$\frac{dG}{dt}=-k_{31}\phi_{1}-k_{32}\phi_{2}$$

(11d) $$\frac{dGn}{dt}=-k_{41}\phi_{1}-k_{42}\phi_{2}$$

(11e) $$\frac{dL}{dt}=k_{52}\phi_{2}$$

(11f) $$\frac{dMAb}{dt}=k_{61}\phi_{1}+k_{63}\phi_{3}$$

Compared to published models such as [3], the number of reactions is reduced. This can be explained by the absence of ammonium and the related overflow mechanism. As our procedure is data-driven, it leads to the identification of the sole phenomena visible in the collected experimental data.

Moreover, our strategy allows to decouple the identification of the stoichiometry from the kinetics or, at least, to get a first estimate of the stoichiometric parameters, independently of the kinetics. This can be an important asset when identifying bioprocess complex models with numerous parameters.

## 5. Parameter Identification

### 5.1. Reaction Rates

Since the double bottleneck glucose-glutamine is reduced to a simple bottleneck depending on both substrates, a reaction rate combining Monod factors is selected

(12a) $$\phi_{1}=\min(\phi_{G},\phi_{G\max})$$

(12b) $$\phi_{2}=\max(0,\phi_{G}-\phi_{G\max})$$

where

(13) $$\phi_{G}=\mu_{\max1}\frac{G}{K_{G}+G}\frac{Gn}{K_{Gn}+Gn}X$$

(14) $$\phi_{G\max}=\mu_{\max2}X$$

while the death rate is given by

(15) $$\phi_{3}=\mu_{d\max}\frac{K_{Gd}}{K_{Gd}+G}\frac{K_{Gnd}}{K_{Gnd}+Gn}X$$

### 5.2. Initial Conditions and Identification Criterion

Starting with the previously obtained values of the stoichiometric matrix $\hat{K}$ in (10a–10c) as stoichiometric parameter initial conditions, the whole parameter set (i.e., stoichiometric and kinetic parameters) can be identified minimizing a least-squares criterion measuring the distance between model simulated data $\xi_{m}$ and experimental measurements $\xi_{\exp}$ as in:

(16) $$J(\theta )={\left(\xi_{m}(\theta )-\xi_{\exp}(\theta )\right)}^{T}Q^{-1}\left(\xi_{m}(\theta )-\xi_{\exp}(\theta )\right)$$

where $\theta =\left[\mu_{\max1}\mu_{\max2}K_{G}K_{Gn}K_{Gd}K_{Gnd}\mu_{d\max}k_{31}k_{41}k_{61}k_{32}k_{42}k_{52}k_{63}\xi_{0}\right]$ is the parameter vector initialized with $\theta_{0}=\left[\mu_{\max1,0}\mu_{\max2,0}K_{G,0}K_{Gn,0}K_{Gd,0}K_{Gnd,0}\mu_{d\max,0}{\hat{k}}_{31}{\hat{k}}_{41}{\hat{k}}_{61}{\hat{k}}_{32}{\hat{k}}_{42}{\hat{k}}_{52}{\hat{k}}_{63}\xi_{0,0}\right]$. The initial state $\xi_{0}$ is a vector of length $N.n_{\exp}$ with $n_{\exp}$ being the number of experiments used in identification. $\xi_{0,0}$ is set using the experimental measurements at time *t* = 0.

Q is the measurement error covariance matrix. As measurement error standard deviations are a priori unknown, it is common choice to set Q to a diagonal matrix with the squares of the maximum respective concentration levels. This allows to normalize the distances calculated in (16) and give equal importance to states with different orders of magnitude. 

Parameter identification is performed with the MATLAB library-optimizer ”fmincon”. This algoritm allows to set box constraints on the parameters so as to limit the search space, and is typically used here in three successive calls. The first call starts from the initial guess (the MLPCA estimates of the stoichiometry, and an “inspired guess” for the kinetics), and the next are initialized with the parameter values resulting from of the previous minimization. Clever initialization is essential in reducing the computational cost and in increasing the chance of capturing the global minimum.

### 5.3. Minimization and Multi-Start Strategy

A multi-start strategy is applied in order to check if convergence is achieved when starting from different locations in the 7-dimensional kinetic parameter space polytope bounded by vertices defined in Table 1, and to identify the best parameter set corresponding to the cost Function (16) global minimum.

25 runs were achieved, leading to the results shown in Table A1 in Appendix A. From a quantitative point of view, the minimization process is achieved efficiently in most of the cases since the cost function residuals are comprised in the interval $J_{res}=\left[\begin{array}{cc} 1.1431 & 1.5686 \end{array}\right]$ in 22 out of the 25 runs (the initial order of magnitude of J is typically between 20 and 100). Runs 11, 16 and 21 lead respectively to $J_{res}=6.7252$, $J_{res}=4.1909$ and $J_{res}=2.8027$, which are highlighted in Table A1 in Appendix A (large deviations in the value of the growth rate are observed). We can conclude that the neighborhood of the optimum should be reached in almost 90% of the runs based on random initialization inside the polytope. 

**Figure 5:**
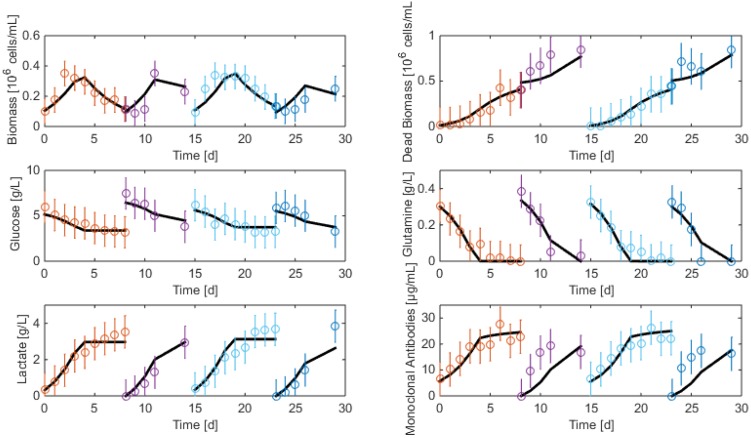
Direct validation of Model (11) on the first two data sets of the HB1 strain. The second experiment starts after 15 days. The solid line represents the model prediction while the circles represent the experimental measurements with a confidence interval at 99%.

Interestingly, all the 22 runs lead to similar direct validation results shown in Figure 5. For the sake of space in this article, both experiments are graphed on the same figure and the second experiment starts after 15 days, i.e. when the first one is over. Overall, the model predicts well the experimental measurements. However, the prediction of antibody concentration is less accurate after the medium renewal at day 8, probably due to inaccurate biomass concentration measurements.

## 6. Parametric Sensitivity Analysis

The evaluation of parametric sensitivities, i.e., the relative influence of the parameters on the model outputs, is useful to assess potential identifiability problems and confidence intervals. Identifiability depends on the model structure and parametrization as well as on the information content of the data. In unfavorable situations a lack of sensitivity could appear or correlation among parameters. When the model is identifiable with the data at hand, sensitivity information can be used to evaluate the Fisher Information Matrix (FIM) and in turn confidence intervals for the several parameters [20].

### 6.1. Parameter Error Covariance

The sensitivity of the *i*th state $\xi_{i}$ with respect to *k*th parameter $\theta_{k}$ at time *t* is theoretically defined by:

(17) $$\xi_{\theta ,i}(t)=\frac{\partial \xi_{i}(t)}{\partial \theta_{k}}$$

Parametric sensitivities can be computed by integration of the following ordinary differential equations:

(18) $${\dot{\xi }}_{\theta ,i}=\frac{\partial f_{i}}{\partial \xi_{i}}\xi_{\theta ,i}+\frac{\partial f_{i}}{\partial \theta }$$

with ${\dot{\xi }}_{i}=f_{i}$ the model state equation.

Parameter identifiability can be assessed using the Fisher Information Matrix (*FIM*), which can be computed as follows

(19) $$FIM=\sum_{t_{k}=1}^{n_{meas}}\xi_{\theta ,i}{\left(t_{k},\hat{\theta }\right)}^{T}Q^{-1}\xi_{\theta ,i}(t_{k},\hat{\theta })$$

where $t_{k}$ is the sampling time and $n_{meas}$ is the number of samples.

An optimistic estimate of the parameter estimation error covariance matrix can be estimated based on the inverse of the *FIM*:

(20) $$\hat{P}>\sigma^{2}FIM^{-1}$$

with $\sigma^{2}$ being the posterior estimate of the measurement error variance obtained from the residual cost function at the optimum:

(21) $$\sigma^{2}=\frac{J^{*?}}{N_{meas}-n_{\theta }}$$

where $N_{meas}$ is the total number of measurements $\left(N_{meas}=n_{meas}N\right)$ and $n_{\theta }$ is the number of estimated parameters.

### 6.2. Application to the Case Study

The relative standard deviations (the square root of the diagonal of (20)) are shown in Table A2 in Appendix A for the 22 optimization runs under consideration. It is apparent that the error on $K_{G}$ is very large, which is a sign that model (11) is over-parameterized. Indeed, glucose concentration levels are low so that $\frac{G}{G+K_{G}}\approx 1$.

## 7. Reduced Model Identification

### 7.1. Model Reduction

Expression (13) is simplified to:

(22) $$\phi_{G}=\mu_{\max1}\frac{Gn}{K_{Gn}+Gn}X$$

Since glutamine is the main nitrogen source dedicated to cell viability, it is not surprising that glutamine becomes responsible of cell growth, i.e. glycolysis and overflow.

### 7.2. Re-Identification

**Figure 6:**
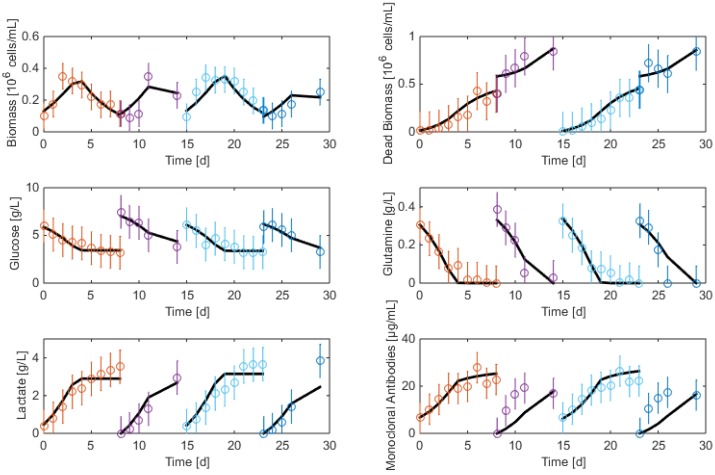
Direct validation of the reduced model with the first two data sets of the HB1 strain. The second experiment starts after 15 days. The solid line represents the model while the circles represent the experimental measurements with a confidence interval at 99%.

With the exception of a few local minima, multi-start identification again leads the minima in the range $J_{res}=\left[\begin{array}{cc} 1.1087 & 1.6534 \end{array}\right]$ and direct validation is shown in Figure 6.

Relative standard deviations are much improved as shown in Table 2, only for the best run (i.e., presenting the best cost function and relative error standard deviations).

### 7.3. Reduced Model Cross-Validation

**Figure 7:**
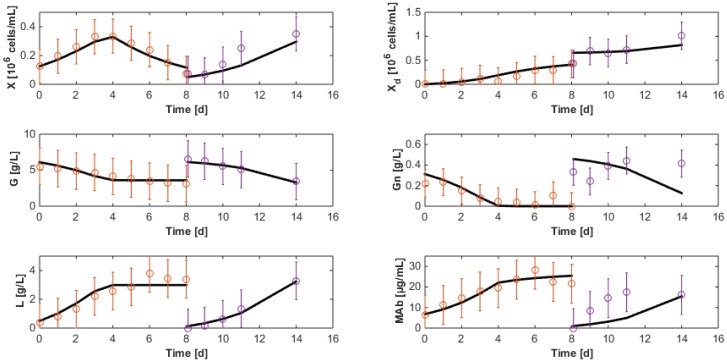
Cross-validation of the reduced model on the third data set of the HB1 strain. The solid line represents the model while the circles represent the experimental measurements with a confidence interval at 99%.

The third data set of HB1 is now used to cross-validate the identified model. During this cross-validation, initial states are re-estimated since initial measurement noise can be a critical source of result degradation. Results shown in Figure 7 are quite satisfactory even though the antibody concentration still suffers from discrepancies after medium renewal. It is worth noticing that the last 3 measurements of glutamine concentration are probably outliers following wrong analytical manipulations (glutamine is only consumed and cannot be produced).

The residual deviation between the model and the experimental data is given by $J_{res}=1.3573$. 

**Figure 8:**
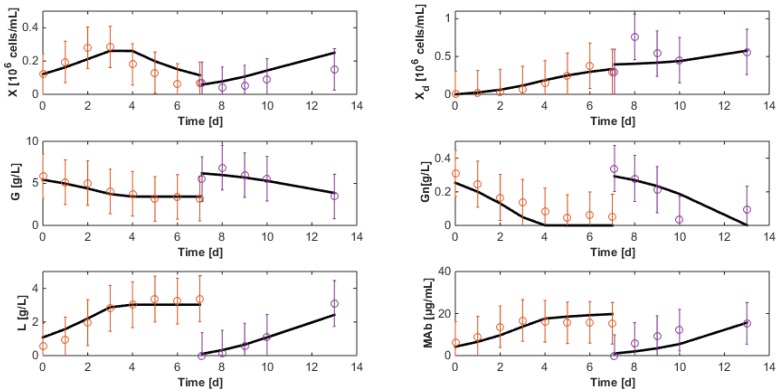
Cross-validation with the second data set of the HB2 strain of the reduced model identified with HB1 data sets. The solid line represents the model while the circles represent the experimental measurements with a confidence interval at 99%.

Interestingly, the model is also able of a relatively good prediction of the experimental data collected with the second hybridoma strain shown in Figure 8. The main discrepancy is in the prediction of the biomass (and consequently of the antibody concentration). However, residuals are still relatively low ($J_{res}=1.4774$), confirming the satisfactory results. These observations allow the perspective that macroscopic models could be adapted from one application to another at relatively little extra costs, just recalibrating the model based on some new available data, starting from the parameter estimates obtained in earlier applications.

### 7.4. Robustness to Parameter Uncertainty

Since the identified parameters of Section 7.2 show some uncertainties represented by their estimation error standard deviations (see Table 2), a Monte-Carlo analysis is developed, where each parameter is subject to normally distributed variations.

**Figure 9:**
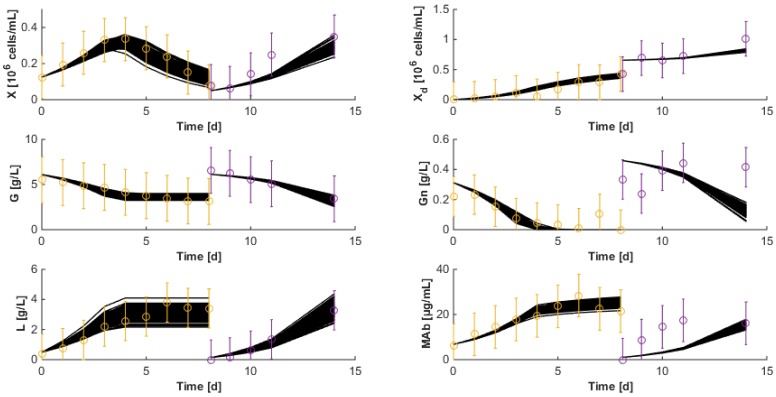
Monte-Carlo analysis of the cross validation of the HB1 model: the solid lines represent 100 model trajectories corresponding to normally distributed parameters characterized by the standard deviations of Table 3. Circles represent the experimental measurements with a confidence interval of 99%.

100 runs of the HB1 model are performed for the HB1 cross-validation data sets and are shown in Figure 9. The trajectory envelope is most of the time contained within the measurement confidence intervals with the exception of the MAb measurements following the medium renewal.

Results of the Monte-Carlo analysis are presented in Table 3.

Parameter variations can have a slight positive effect on cross validation (since the residual cost function was initially 1.3573 and the best Monte-Carlo case is 1.3412) but usually a negative effect, the worst case corresponding to a residual cost of 1.6225. Since all the runs provide satisfactory results, the identified model is quite acceptable for prediction and control purposes. 

## 8. Optimization of the Monoclonal Antibody Production

This section intends to provide the best medium renewal time and composition to maximize the monoclonal antibody production and substrate savings. Using the validated model of Section 7, it is possible to express these targets in a mathematical objective function of the form:

(23) $$J_{obj}=MAb{\left(t_{renewal}\right)}^{2}+MAb{\left(t_{f}\right)}^{2}-\alpha (G_{renewal}^{2}+Gn_{renewal}^{2})$$

where α represents a weighting coefficient penalizing substrate savings with respect to MAb production, i.e. defining the degree of predominance of one target over the other. Minimization of (25) is achieved using the optimizer *fmincon* from the Matlab platform, in order to find the best values of θ = [t_renewal_ G_renewal_ Gn_refresh_], i.e., is the medium renewal time t_renewal_ and the glucose and glutamine concentrations in the medium G_renewal_ and Gn_renewal_. *Fmincon* also allows to specify box constraints for t_renewal_ Є [3 14] days. These values are selected in accordance with the previous experimental results: medium renewal should on the one hand not occur too soon and on the other hand before the end of the experiment set at day 14. G_renewal_ Є [1 15] g/L and Gn_renewal_ Є [0.1 1] g/L so as to avoid cell starvation or growth inhibition through the accumulation of byproduct.

**Figure 10:**
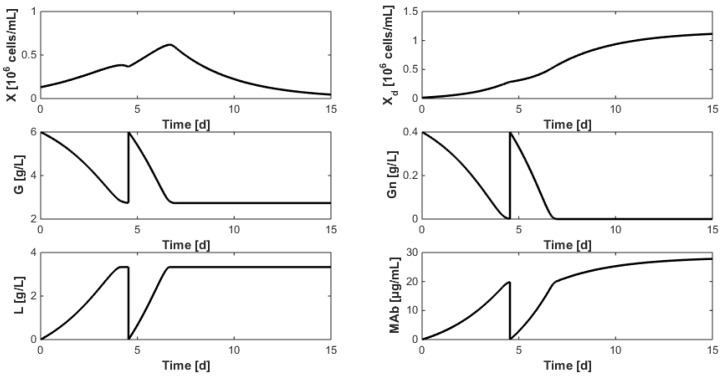
Optimization of the renewal time with medium substrate concentrations respectively set to G = 6 g/L and Gn = 0.4 g/L (α = 0), while maximizing monoclonal antibody production.

Figure 10 shows the optimization results when α is set to zero and G_renewal_ and Gn_renewal_ are respectively set to 6 and 0.4 g/L (which is similar to the concentrations used in the experiments dedicated to model identification described in Section 7). The best time at which medium renewal should be achieved is found at t_renewal_ = 4.54 days (in the previous experiments, renewal had been achieved after approximately 7 days). Moreover, the MAb production, defined as the sum of the final batch concentrations, amounts to 60.92 µg/mL which represents a production gain of 30% with respect to the experiments of Figure 1 (where the production can be estimated to 40 to 45 µg/mL).

**Figure 11:**
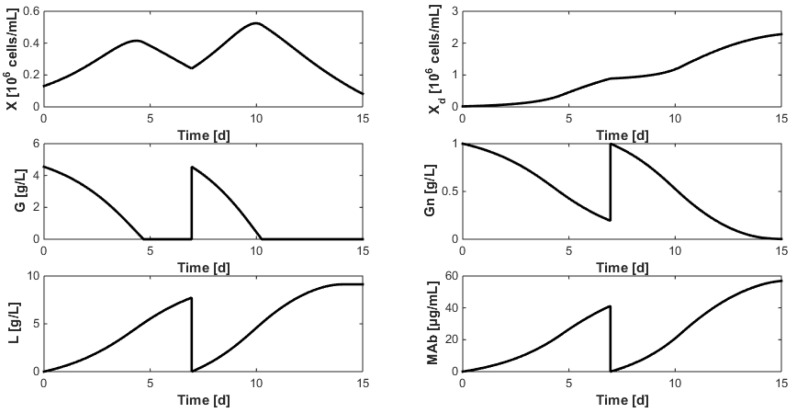
Optimization of the renewal time when an important substrate saving (α = 10) to maximize the produced monoclonal antibody amount.

Figure 11 shows new results when a strong emphasis is placed on substrate savings with α = 10. The optimizer converges to θ = [6.95 4.55 1], which leads to the following observations:

- Even when considering substrate savings, the upper bound of Gn_refresh_ is reached. Indeed, when G is depleted, Gn still limits biomass decay and therefore maintains an efficient MAb production rate. However, since ammonium production (byproduct formed by glutamine overflow) is not considered in the model obtained in Section 7, higher values of Gn_refresh_ are not recommended.
- Interestingly, approximately the same renewal time as in Figure 2 is obtained, which means that these experiments could be “economically” optimized only by revising the medium composition.

**Figure 12:**
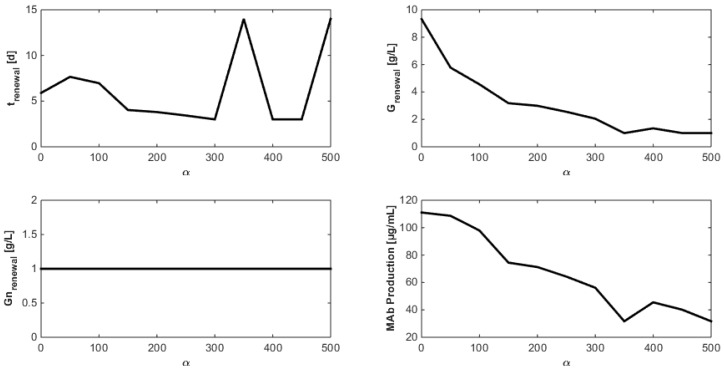
Evolution of the culture operating conditions and MAb production as functions of α.

Since MAb production clearly appears as a function of substrate saving penalization, new optimizations considering α in the range 0 to 500 with incremental steps of 50 are achieved in order to assess the impact of α on MAb production and select a good compromise. Results displayed in Figure 12 show that specific operating conditions can be chosen to reach a target MAb production. For instance, approximately 3 g/L of glucose are sufficient, with a renewal after 4 days, to harvest 75 µg/mL of MAb within 14 days. Moreover, operating conditions of Figure 2 seem to be a good economic compromise as 100 µg/mL can be harvested starting with a glucose concentration of 6 g/L and a renewal after 7 days. Concerning the glutamine concentration, the observations from Figure 10 are confirmed: since no glutamine overflow is considered, very high glutamine concentrations are unrealistically tolerated. 

## 9. Conclusions

In this work, a simple dynamic model of hybridoma sequential batch cultures is developed, which can be used to optimize the production of monoclonal antibodies. 

Maximum likelihood principal component analysis allows assessing the information content of the experimental data, providing the minimum number of reactions and the corresponding stoichiometry by solving an optimization problem under a few a priori biological constraints. An original formulation of the method is presented, allowing biomass to occur in several reactions. 

Advantages of the method are (a) to limit the number of reactions, i.e. to avoid a useless complication of the model with respect to the experimental field and the involved biological phenomena (activation, saturation, inhibition, etc.); (b) to offer the possibility to proceed to a quick first estimation of the stoichiometry independently of the kinetics, in turn reducing the number of unknown parameters (for the current model, stoichiometry represents half the parameter set); (c) to provide a “divide and conquer” approach where the stoichiometry and kinetics can be estimated separately or simultaneously, in an iterative way, starting from estimates obtained at the previous step.

The procedure can be supplemented by parametric sensitivity analysis, which allows further model simplification, whenever needed, by isolating parameters with low sensitivities.

A Monte-Carlo study, where parameter variations are considered in accordance with the resulting estimation error variances, shows that model trajectories are globally kept inside a corridor defined by measurement confidence intervals (i.e., parameter discrepancies do not cause critical model misevaluations). 

As a practical illustrative outcome of the present study, the obtained dynamic model is used for a two-sequential batch process optimization (determination of the best sequence and composition of medium renewals). The results show that the importance of substrate savings drives the location of the optimum. A renewal time scheduling can therefore be established based on the user will to save medium components such as substrates. Further experimental validations of the optimization method are important perspectives of this still on-going work as well as estimation and on-line control issues.

## Figures and Tables

**Table 1:** Vertices of the multi-start parameter polytope.

Kinetic Parameters/Min–Max Values	Minimum Initial Value	Maximum Initial Value
$\mu_{\max1}$	0.1 h^−1^	1 h^−1^
$\mu_{\max2}$	0.1 h^−1^	1 h^−1^
$K_{G}$	0.01 g/L	1 g/L
$K_{Gn}$	0.01 g/L	1 g/L
$K_{Gd}$	0.1 g/L	10 g/L
$K_{Gnd}$	0.1 g/L	10 g/L
$\mu_{d\max}$	0.1 h^−1^	1 h^−1^

**Table 2:** Parameter estimates and relative estimation error for the best identification run of the reduced model.

Parameter		Relative Error Standard Deviation
$\mu_{\max1}$	0.4849	1.7652
$\mu_{\max2}$	0.3198	8.1283
$K_{Gn}$	0.0089	23.2540
$K_{Gd}$	1.5899	15.4666
$K_{Gnd}$	1.3359	49.2893
$\mu_{d\max}$	0.8667	66.5216
$k_{31}$	3.1207	34.1265
$k_{32}$	15.2090	26.3604
$k_{41}$	0.6245	9.5667
$k_{42}$	1.2221	19.2377
$k_{52}$	23.9586	20.3273
$k_{61}$	43.5907	8.3527
$k_{63}$	14.2221	10.9999

**Table 3:** Results of the Monte-Carlo analysis: number of runs, minimum, maximum and mean values of the residual cost function *J* and standard deviation.

Runs	Min J	Max J	J Mean	J Std Deviation
100	1.3412	1.6225	1.4050	0.0576

## Appendix A

**Table A1:** Parameter identification following the 25 multi-start runs (orange lines indicate clear local minima).

$\mu_{\max1}$	$\mu_{\max2}$	$K_{G}$	$K_{Gn}$	$K_{Gd}$	$K_{Gnd}$	$\mu_{d\max}$	$k_{31}$	$k_{32}$	$k_{41}$	$k_{42}$	$k_{52}$	$k_{61}$	$k_{63}$
0.5559	0.1410	0.1456	0.0224	2.1895	0.1922	0.7028	2.4175	6.0250	1.2867	0.5061	10.6769	119.7005	7.6599
0.5880	0.2670	0.0451	0.0162	1.9315	1.1321	0.7073	2.3867	6.6606	0.7739	0.6705	14.6022	68.0054	7.7126
0.5547	0.1500	0.0115	0.0094	1.2845	1.2627	0.9401	4.2541	4.4612	0.4948	0.8604	9.6828	114.3043	9.5609
0.5473	0.1429	0.0280	0.0145	1.9152	0.3041	0.7235	2.8453	6.0009	0.7705	0.7854	10.3002	122.0962	10.2667
0.6058	0.0611	0.0526	0.0413	0.8423	0.9630	1.2922	4.2558	5.7751	2.0796	0.5338	8.1830	234.9037	14.3253
0.5626	0.1100	0.0521	0.0286	0.9322	0.3854	1.2512	3.2340	5.6626	1.5257	0.4498	9.6220	161.7081	4.3626
0.5887	0.2774	0.0273	0.0170	2.3108	1.7476	0.6160	2.7446	8.1550	0.8308	0.5936	15.0889	62.0973	10.5890
0.6303	0.1737	0.0988	0.0229	2.0640	2.0451	0.6517	2.7454	6.1184	1.0883	0.5328	10.1684	94.7350	13.3752
0.6139	0.2062	0.1491	0.0195	1.0761	1.6163	1.0645	2.8275	6.4991	0.9660	0.5718	11.4731	78.7716	13.9319
0.5195	0.2916	0.0223	0.0061	2.5413	0.3398	0.6242	3.9897	5.4858	0.3507	1.5180	18.3348	65.0805	5.7034
0.2676	0.5515	0.0170	0.0123	1.8476	1.9618	0.1667	3.5684	13.6956	1.4072	1.3057	15.4667	107.3452	17.9771
0.6048	0.1698	0.0305	0.0232	2.0572	0.7868	0.6622	3.5670	5.3198	1.1648	0.5179	10.4758	104.5675	9.7748
0.5868	0.1808	0.0329	0.0219	2.4127	1.7841	0.6059	3.3243	5.3469	1.0981	0.4273	10.7470	94.6231	7.8496
0.5865	0.1951	0.0285	0.0185	1.1563	0.6525	1.0732	2.5100	4.3891	0.8773	0.6274	11.3024	98.2351	3.4642
0.5287	0.2558	0.0744	0.0099	2.4822	2.1080	0.6520	5.1365	4.3431	0.6706	0.8310	16.4441	59.0160	12.3303
0.9859	0.6572	0.0100	0.1800	5.7454	2.3415	0.3338	7.0345	0.2045	0.8892	0.0953	0.0064	50.4414	0.0511
0.5609	0.2443	0.0260	0.0116	0.8086	0.6322	1.3976	2.7069	6.8815	0.5485	0.9639	13.8020	76.5734	6.3556
0.6099	0.0984	0.1118	0.0176	2.1902	1.6137	0.6266	2.4999	5.1055	0.8327	0.7344	8.4616	146.6756	18.1001
0.6311	0.1634	0.0090	0.0452	1.2904	1.3282	0.8407	0.5745	9.7854	1.9713	0.1358	10.8829	126.9890	0.2194
0.5854	0.1725	0.1799	0.0251	1.7937	0.2995	0.7516	2.8422	6.3408	1.2724	0.4102	11.3594	101.7783	8.0611
0.3523	0.1682	0.2644	0.0147	0.2683	0.2306	0.3314	3.5112	17.5043	0.9900	1.5072	27.5654	98.4703	16.7248
0.5577	0.3183	0.0328	0.0073	1.6475	1.3849	0.8834	2.8735	5.5942	0.4435	1.1310	17.5563	6.7974	47.0452
0.6057	0.2115	0.0757	0.0163	1.5704	1.5814	0.8071	2.4129	5.6236	0.7583	0.6688	11.0633	87.1816	7.4376
0.5954	0.1964	0.0197	0.0171	1.2385	0.7389	0.9992	2.3813	5.4283	0.7576	0.7362	11.0359	100.4704	3.6202
0.5482	0.2715	0.0400	0.0182	1.5790	0.2627	0.8576	6.9704	1.5800	1.0154	0.3502	17.0623	67.7835	6.2548

**Table A2:** Parameter estimation error relative standard deviations for the 22 selected runs.

$\mu_{\max1}$	$\mu_{\max2}$	$K_{G}$	$K_{Gn}$	$K_{Gd}$	$K_{Gnd}$	$\mu_{d\max}$	$k_{31}$	$k_{32}$	$k_{41}$	$k_{42}$	$k_{52}$	$k_{61}$	$k_{63}$
3.6662	22.6437	130.2128	17.8640	49.5212	13.2501	31.2599	120.2970	24.7702	21.8237	16.9119	10.8063	22.3173	22.0970
3.1545	11.4168	361.2676	18.2503	55.3056	40.4276	36.8463	57.3265	26.6477	11.5497	15.0769	12.9871	11.3732	22.5188
2.7529	27.1667	1181.2541	23.8344	46.7243	43.2328	34.3950	73.5869	32.0299	30.7479	11.1012	12.1905	27.1262	20.1369
3.6238	24.3889	720.1689	20.7425	59.3874	18.0344	39.2357	103.7392	24.0107	24.4068	11.3759	11.0635	24.1557	17.9644
5.1282	46.0975	628.3497	18.5780	114.7653	46.7917	93.0397	126.2801	16.7504	42.9210	10.6211	8.0060	45.4639	15.6957
3.6607	27.9484	379.5510	17.5197	73.0553	20.8103	57.9583	107.1231	22.6208	26.9998	16.0283	9.7616	27.6345	44.1384
3.0754	11.0214	574.9346	18.7735	51.3645	61.7058	31.3976	48.8369	23.7644	10.9953	17.3372	13.4790	10.9730	16.2965
3.7369	19.7116	211.5824	17.6190	54.5606	71.9202	34.9982	83.7420	21.9111	18.2301	14.1168	10.5920	19.4313	13.2615
3.8635	16.5377	145.3122	18.6602	84.8642	60.1199	65.9035	69.0624	23.7561	15.6449	15.0802	11.7848	16.3232	12.8728
2.5121	10.8171	546.0726	24.6565	49.0097	18.0168	29.2449	32.5452	39.8743	17.6116	15.2724	16.7534	10.9220	30.5888
3.5450	19.6658	640.1403	17.3780	54.9197	32.1188	35.3471	67.2044	25.6640	18.4603	15.0928	10.6327	19.3963	17.6762
3.9618	18.6565	667.0940	18.3143	57.4314	68.1850	34.5779	65.4403	26.6676	17.9300	18.1388	11.5034	18.5005	24.8874
3.4707	16.9881	644.5080	17.9580	71.5894	26.5994	55.1697	79.9299	32.5613	16.7107	13.7367	11.4200	16.8977	52.9654
4.2695	14.5552	280.6340	24.0336	59.1986	67.5103	36.0739	34.9784	50.1691	15.7086	18.0133	17.5370	14.6961	16.8627
3.0381	13.3080	609.3884	20.3656	87.6372	27.4640	72.0226	57.3727	25.6625	15.4098	12.4396	13.1777	13.3089	27.3362
3.7332	36.3680	186.6357	20.3580	49.4555	58.1809	31.1673	159.1482	21.3178	32.7821	9.1231	9.4398	35.9587	11.4881
3.3887	16.9274	1933.8346	15.1813	69.0932	49.6417	50.7343	401.5180	14.7875	16.6962	52.5206	9.3112	16.6627	722.5528
3.5640	17.3184	110.6870	17.5278	61.0394	17.2553	41.0509	77.7436	23.3178	16.9096	19.8023	10.6291	17.0292	21.2591
3.2513	12.6435	484.7580	27.1346	58.2372	46.5384	40.9441	46.2395	44.1952	15.8306	17.7920	20.7195	25.5029	4.2209
3.4716	16.2920	247.1369	18.5861	60.9806	55.8643	43.1792	76.6727	26.1026	16.0291	12.9251	11.8084	16.2252	24.4574
3.2776	16.8715	871.4772	17.9683	61.5156	28.8860	46.3341	82.1495	25.7187	16.8224	11.7135	11.0761	16.8066	49.3217
3.0620	10.1596	397.7225	18.8584	77.1272	16.8420	54.2770	20.9435	121.3063	10.1811	31.2955	13.8143	9.9632	24.4122
